# Nanocellulose-stabilized Pickering emulsions and their applications

**DOI:** 10.1080/14686996.2017.1401423

**Published:** 2017-11-23

**Authors:** Shuji Fujisawa, Eiji Togawa, Katsushi Kuroda

**Affiliations:** ^a^ Forestry and Forest Products Research Institute, Tsukuba, Japan

**Keywords:** Pickering emulsion, nanocellulose, double emulsion, surface modification, 20 Organic and soft materials (colloids, liquid crystals, gel, polymers), 212 Surface and interfaces

## Abstract

Pickering emulsion, which is an emulsion stabilized by solid particles, offers a wide range of potential applications because it generally provides a more stable system than surfactant-stabilized emulsion. Among various solid stabilizers, nanocellulose may open up new opportunities for future Pickering emulsions owing to its unique nanosizes, amphiphilicity, and other favorable properties (e.g. chemical stability, biodegradability, biocompatibility, and renewability). In this review, the preparation and properties of nanocellulose-stabilized Pickering emulsions are summarized. We also provide future perspectives on their applications, such as drug delivery, food, and composite materials.

## Graphical abstract

**Figure:**
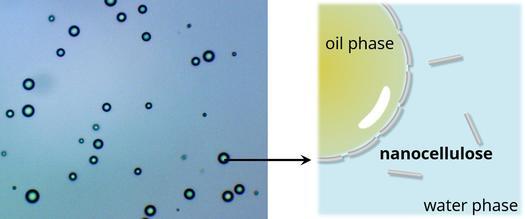

## 1. Introduction

An emulsion is a system consisting of two immiscible liquids, in which droplets of one liquid is dispersed in another. The system is optically isotropic, typically forming nano- or micron-ordered droplets throughout the system. Hence, it offers remarkable potential for applications that need macroscopically homogeneous mixtures or efficient delivery systems (e.g. pharmaceuticals [1–3], cosmetics [3–5], food [6–8], fuel [9,10], and templates for other materials, such as porous material [11,12], liquid foam and emulsion films [13], and electrospun core–shell nanofibers and hollow nanotubes [14]). However, these nano- and micro-emulsions show poor stability in most media due to their large interfacial areas. Therefore, surfactants are typically added to stabilize the system; surfactants preferentially adsorb at immiscible liquid/liquid interfaces due to their amphiphilic properties, and suitable selection of surfactants leads to efficient reduction in the interfacial energy [14].

**Figure 1.**
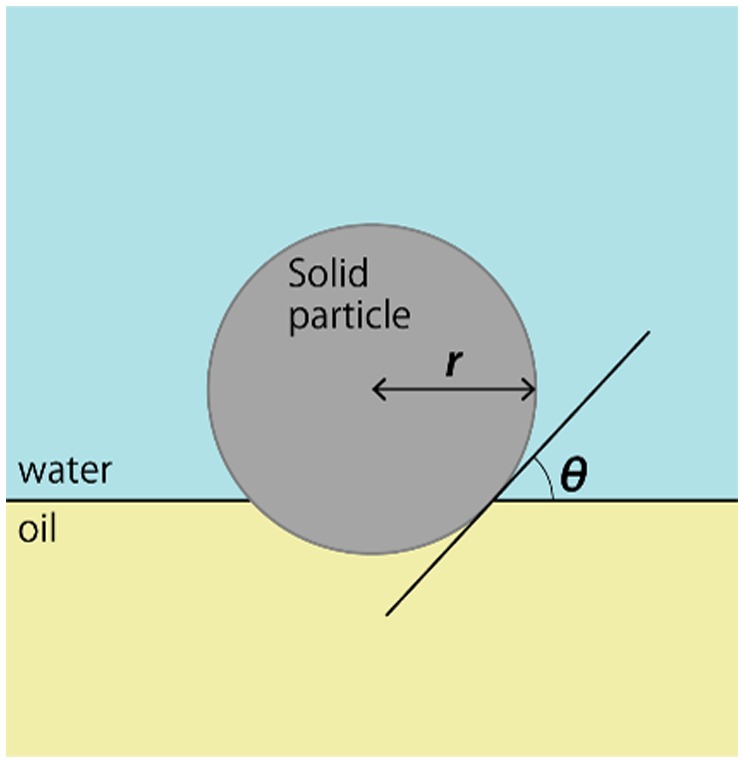
Solid particle at a planar oil/water interface.

Solid fine particles have great potential as emulsion stabilizing agents. This type of emulsion is called Pickering emulsion [15–21]. Unlike surfactant molecules, the particles irreversibly adsorb at liquid/liquid interfaces due to their high energy of adsorption, and therefore, the Pickering emulsion generally form more stable emulsion system than that stabilized by surfactants, which could provide great versatility in material processing [22]. Equation 1 shows the change in interfacial energy, Δ*E*, when a solid sphere is adsorbed at the interface with a contact angle, *θ,*

(1) $$\Delta E=-\pi r^{2}\gamma_{\text{ow}}{\left(1\pm \text{cos}\theta \right)}^{2}$$

where *r* and *γ*_ow_ represent the radius of the particle and oil/water interfacial tension, respectively (see Figure 1). When *θ* < 90°, the particle is relatively hydrophilic and the sign inside the parenthesis is negative, and when *θ* > 90°, it is positive. As described by Equation 1, the adsorption is the strongest when *θ* = 90°. The particles are strongly adsorbed at the interface, and the energy required to desorb the particle from the interface, or –Δ*E*, is orders of magnitude higher than that of soluble surfactants [17]. Various solid particles have been used as stabilizers for Pickering emulsions. Previous studies have dealt with fine organic or inorganic nanomaterials, including graphene oxide [23], carbon nanotube [24], carbon lamp black [25, 26], laponite [27–29], montmorillonite [30], silica nanoparticles [31–33], calcium carbonate (CaCO_3_) [34], titanium dioxide (TiO_2_) [35], magnetic particles [36, 37], and polymer particles [38–40]. Surface modification can tailor the wettability of these fine particles [19], which can effectively change the emulsion phase between oil-in-water (o/w) and water-in-oil (w/o) emulsions (continuous phases are water and oil, respectively). These unique characteristics and variability of Pickering emulsions may open up new opportunities for future emulsion-based materials.

Nanocellulose satisfies the increasing demands for a sustainable and environmentally friendly stabilizer for Pickering emulsions; nanocelluloses efficiently stabilize oil/water interfaces due to their amphiphilic surface nature, which originates from the hydrophobic face and hydrophilic edge of cellulose chains [41–43]. Nanocelluloses are typically prepared through mechanical disintegration of bundles of cellulose microfibrils. The width, length, and surface properties of nanocellulose can be controlled by changes in cellulose sources and pretreatment: mechanical treatment only [44–47] or with pretreatment, such as acid hydrolysis [48, 49], 2,2,6,6-tetramethylpiperidine-1-oxyl (TEMPO)-mediated oxidation [50–52], carboxymethylation [53], phosphorylation [54, 55], and enzymatic hydrolysis [56–59]. Given that the original crystal structure of cellulose microfibrils [60–62] remains intact even after preparation, nanocelluloses have high modulus (130–150 GPa) [63–65] and strength (2–6 GPa) [66, 67] and low coefficient of thermal expansion (4–6 ppm K^−1^) [68, 69] along the *c*-axis. These excellent mechanical properties play an important role in structurally stabilizing the interfaces. Moreover, surface modification of nanocelluloses can tailor wettability at the oil/water interfaces.

This review summarizes recent advances in the Pickering emulsions stabilized with not only nanocelluloses, such as cellulose nanocrystals (CNCs), microfibrillated celluloses (MFCs), bacterial cellulose nanofibrils (BCNs), and TEMPO-oxidized nanocelluloses, but also microcrystalline cellulose (MCC). First, we outline several approaches for the preparation of the emulsions. Second, we introduce materials based on the emulsions and discuss their potential applications, such as drug delivery, food, and composite materials.

## 2. Formation of nanocellulose-stabilized Pickering emulsion

### 2.1. Oil-in-water (o/w) emulsion

**Figure 2.**
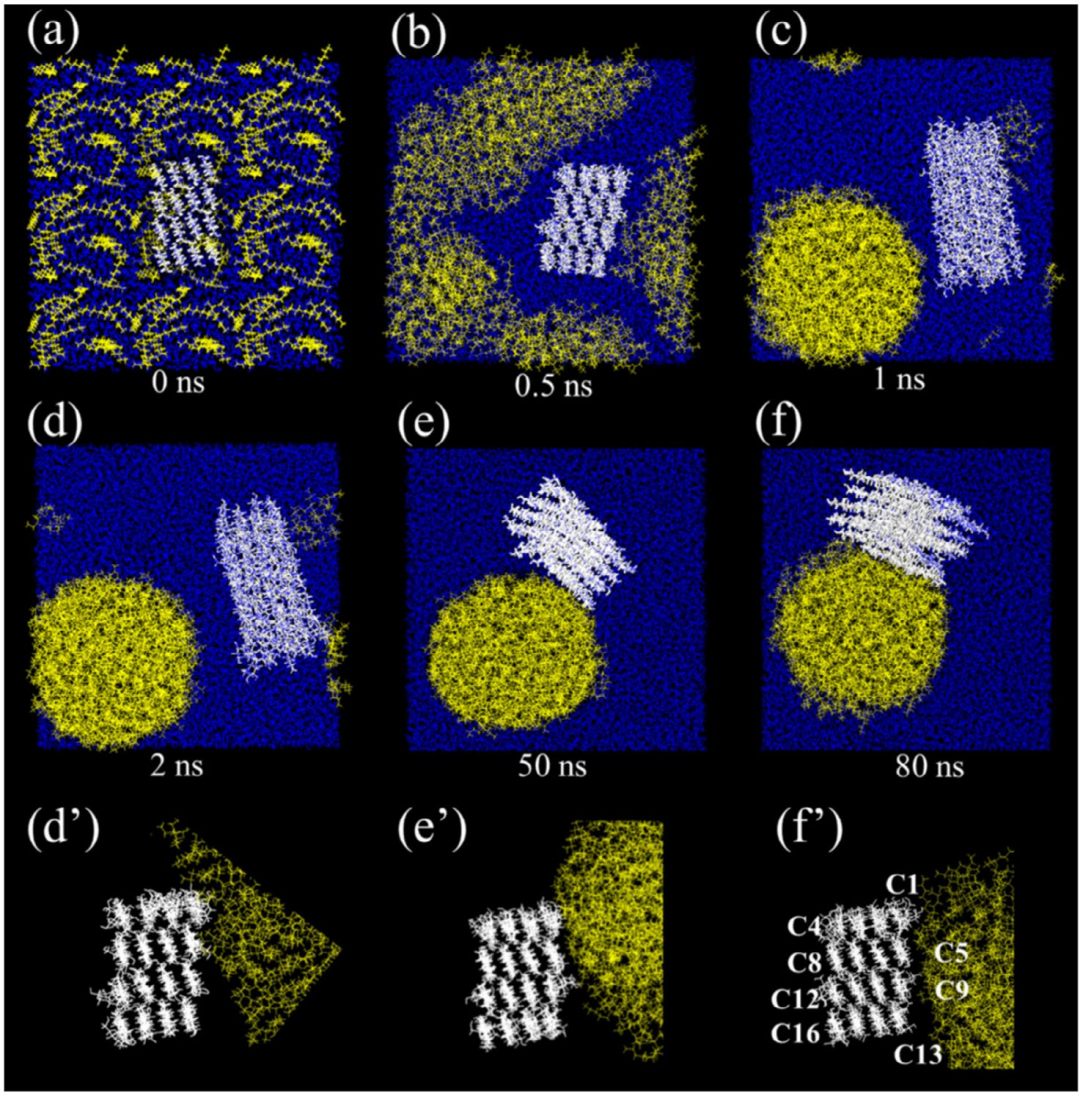
Snapshots of the behavior of separated cellulose chains in the oil-in-water medium during molecular dynamics simulation. The molecules are drawn using the stick representation for cellulose (white), and the line representation for water (blue) and octane (yellow). (Color figure online). Modified from Ref. [83], with permission from Springer (© Springer 2017).

**Figure 3.**
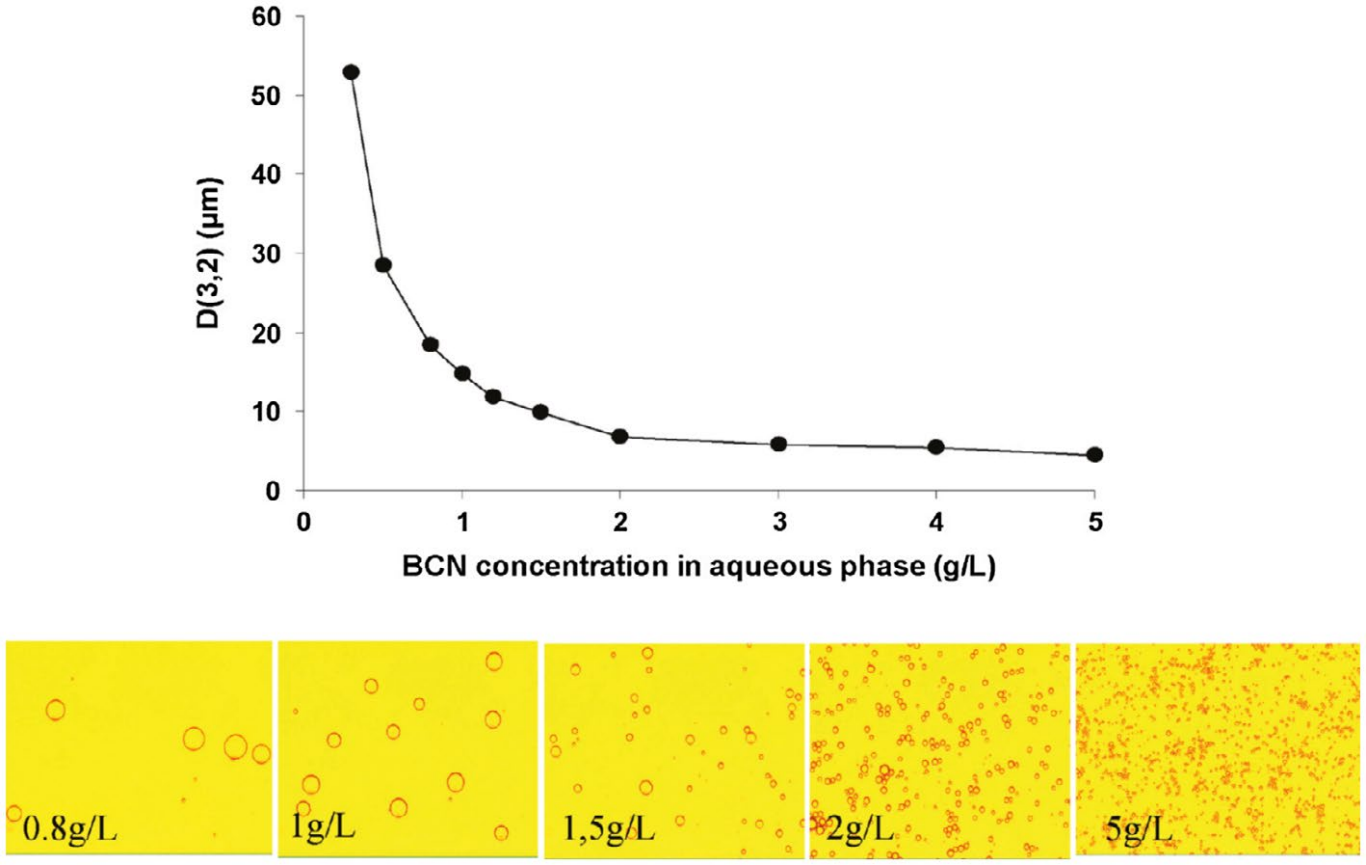
Droplet size dependence on bacterial cellulose nanofibril (BCN) content (upper figure) for droplet diameters D(3,2) versus BCN concentration in the water phase in an emulsion containing hexadecane with a 30/70 oil/water ratio. (lower images) Transmission optical micrographs of the same emulsions; the corresponding concentrations are given in the images. Modified from Ref. [79], with permission from American Chemical Society (© ACS 2011).

Nanocelluloses are likely to form o/w emulsions, and the emulsions are typically prepared by mechanical treatment of the mixture of oil and nanocellulose aqueous dispersion, in which nanocelluloses are adsorbed at the oil/water interfaces [41] and stabilize the emulsion. The ability to form the Pickering emulsions is not significantly different among cellulose sources, such as wood cellulose [70–74], cotton [41, 75–78], bacterial cellulose [41, 73, 77, 79], *Cladophora* [77], and other plants [80, 81] (see Table 1) because native celluloses have common crystal allomorphs, collectively called cellulose I. A hydrophobic edge plane appears responsible for the wettability of CNCs at the oil/water interface [41], and this observation was also explained by molecular modeling (Figure 2) [82, 83]. Nanocellulose-stabilized Pickering emulsions exhibit good stability against coalescence. Kalashnikova et al. reported that hexadecane droplets around 4 μm in diameter are stable for several months (Figure 3) and resistant to centrifugation at 4000 *g* [79]. Nanocellulose-stabilized emulsions are generally stable to heating in a wide temperature range owing to their structural stability. When nanocelluloses without any surface charges are used as stabilizers, the emulsion formed shows good stability against salt and pH changes [73].

**Table 1.** Summary of cellulose-stabilized Pickering emulsions.

Type	Cellulose source	Cellulose [surface modification]	Disperse phase	Continuous phase	Reference
o/w	Bacterial cellulose	CNC	Hexadecane, Styrene	Water	Kalashnikova et al. [79]
	Bacterial cellulose, Cotton	CNC	Hexadecane, Styrene	Water	Kalashnikova et al. [41]
	Cotton	CNC	Styrene	Water	Nypelö et al. [75]
	Cotton	CNC	Hexadecane	Water	Capron and Cathala [76]
	Corncob	CNC	_D_-limonene	Water	Wen et al. [81]
	Cotton, Bacterial cellulose, *Cladophora*	CNC	Hexadecane, Styrene	Water	Kalashnikova et al. [77]
	Softwood sulfite pulp, Cotton	CNC, NFC	Hexadecane	Water	Cunha et al. [78]
	Wood pulp	CNC, NFC, TOCN	Dodecane	Water	Gestranius et al. [70]
	Wood pulp	CNF/NFC	Hexadecane/IPDI/DBDL	Water	Svagan et al. [71]
	Wood cellulose	MCC	Sunflower oil	Water	Kargar et al. [72]
	Bacterial cellulose, Wood cellulose	BCN, MCC, MFC	Vegetable oil, Kerosene	Water	Ougiya et al. [73]
	Mangosteen rind	MFC	Soybean oil	Water	Winuprasith and Suphantharika [80]
	Softwood sulfite pulp	TOCN	Paraffin	Water	Li et al. [82]
	Wood pulp	TOCN	Styrene	Water	Fujisawa et al. [74]
	Cotton	CNC [VAc, VCin]	Ethyl acetate, Toluene, Cyclohexane	Water	Sèbe et al. [84]
	Cotton	CNC [CTAB, DMAB]	Dodecane	Water	Hu et al. [91]
	Ramie fiber	CNC [Poly(NIPAM)]	Heptane	Water	Zoppe et al. [85]
	Wood pulp	CNC [PDMAEMA]	Heptane, Toluene	Water	Tang et al. [86]
					
w/o	Cotton	CNC [CTAB, DMAB]	Water	Dodecane	Hu et al. [91]
	Cotton, Softwood sulfite pulp	NFC, CNC [Lauroyl chloride]	Water	Hexadecane	Cunha et al. [78]
	Softwood sulfite pulp	MFC [CDMIPS]	Water	Toluene	Andresen and Stenius [87]
	Softwood pulp	MFC [CDMIPS]	Water	Toluene	Xhanari et al. [88]
	Softwood pulp	MFC [Octadecylamine, Poly(St-*co*-MA)]	Water	FT-diesel	Lif et al. [89]
	Bacterial cellulose	BCN [AA, HA, DA]	Water	Toluene	Lee et al. [90]

Notes:

CNC: cellulose nanocrystal. NFC: nanofibrillated cellulose. TOCN: TEMPO-oxidized cellulose nanofibril. MCC: microcrystalline cellulose. MFC: microfibrillated cellulose. BCN: bacterial cellulose nanofibrils. IPDI: Isophorone diisocyanate. DBDI: dibutyltin dilaurate. VAc: vinyl acetate. VCin: vinyl cinnamate. CTAB: cetyltrimethylammonium bromide. DMAB: didecyldimethylammonium bromide. Poly(NIPAM): Poly(*N*-isopropylacrylamide). PDMAEMA: poly[2-(dimethylamino)ethyl methacrylate]. CDMIPS: chlorodimethyl isopropylsilane. Poly(St-co-MA): Poly(styrene-co-maleic anhydride). FT-diesel: Fischer–Tropsch diesel. AA: acetic acid. HA: hexanoic acid. DA: dodecanoic acid.

**Figure 4.**
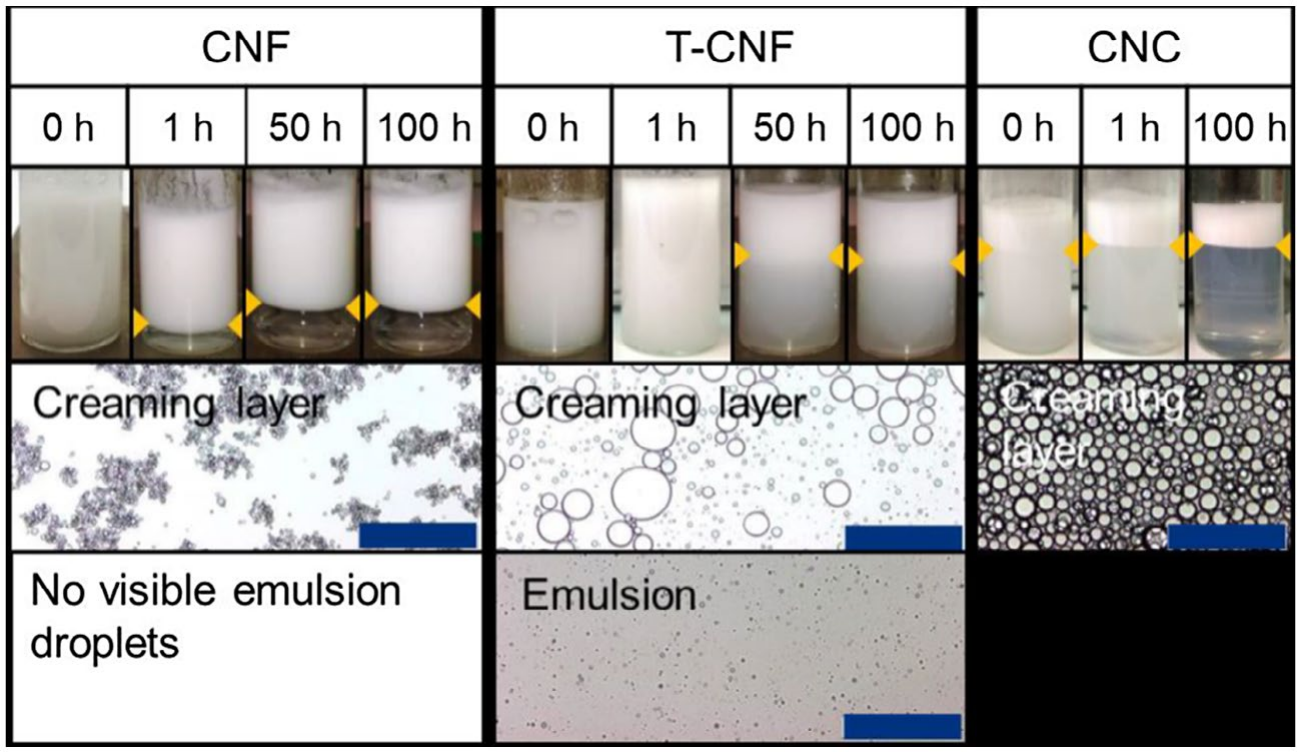
Phase behavior of nanocellulose-stabilized emulsions as a function of time. The samples in the images contain 1% nanocellulosic material and 20% oil, and the creaming layer is indicated with orange arrows. Samples for micrographs of the different phases were transferred to separate vials at 24 h after preparation, and images were taken at 72 h after preparation. The scale bar is 200 μm. Modified from Ref. [70], with permission from Elsevier (© Elsevier 2017).

Surface charges of nanocelluloses play an important role in the stability of the emulsions. When surface-carboxylated nanocelluloses that are prepared by TEMPO-mediated oxidation are used as stabilizers, the nanocelluloses form stable emulsions [74]. The repulsive forces between the droplets arise primarily from osmotic pressure caused by the carboxyl groups. Gestranius et al. compared the phase behavior of the emulsions between the nanocelluloses with or without surface carboxyl groups, and they reported that the surface charges effectively enhance stability (Figure 4) [70]. Kalashnikova et al. reported that CNCs with a surface charge density above 0.03 e/nm^2^ are unable to efficiently stabilize at the oil/water interface, regardless of cellulose sources [41].

Kalashnikova et al. found that aspect ratios of CNCs influence the behavior of emulsion formation [77]; densely covered emulsion surfaces are formed by short nanocrystals, whereas low covered surfaces are formed by long CNCs, and the aspect ratios range from 13 to 160. They estimated that a dense interconnected network is promoted when long CNCs are used, thereby leading to stability at low concentrations. By increasing the nanocellulose concentration, stability to creaming is significantly improved due to the dense network structure [80].

Even after some surface modifications, the modified nanocelluloses can form o/w emulsions. CNCs esterified by vinyl acetate form ethyl acetate-in-water, toluene-in-water, and cyclohexane-in-water emulsion systems, whereas CNCs esterified by vinyl cinnamate stabilize only cyclohexane-in-water emulsions [84]. Therefore, phase behavior of the nanocellulose-stabilized Pickering emulsions can be changed by controlling the surface properties. When poly(*N*-isopropylacrylamide) (poly(NIPAM)) chains are grafted onto CNC surfaces, the formed emulsion shows no phase separation and is stable for more than 4 months, whereas unmodified CNCs are unable to stabilize the emulsion system [85]. The emulsions are stable at ambient conditions, but they break after heating at a temperature above the lower critical solution temperature of poly(NIPAM). pH-responsive CNC-stabilized Pickering emulsions can be formed by grafting a weak polyelectrolyte poly[2-(dimethylamino) ethyl methacrylate] (PDMAEMA) [86]. Chain conformation of the PDMAEMA chains varies with pH and triggers the emulsification and demulsification of oil droplets.

### 2.2. Water-in-oil (w/o) emulsion

**Figure 5.**
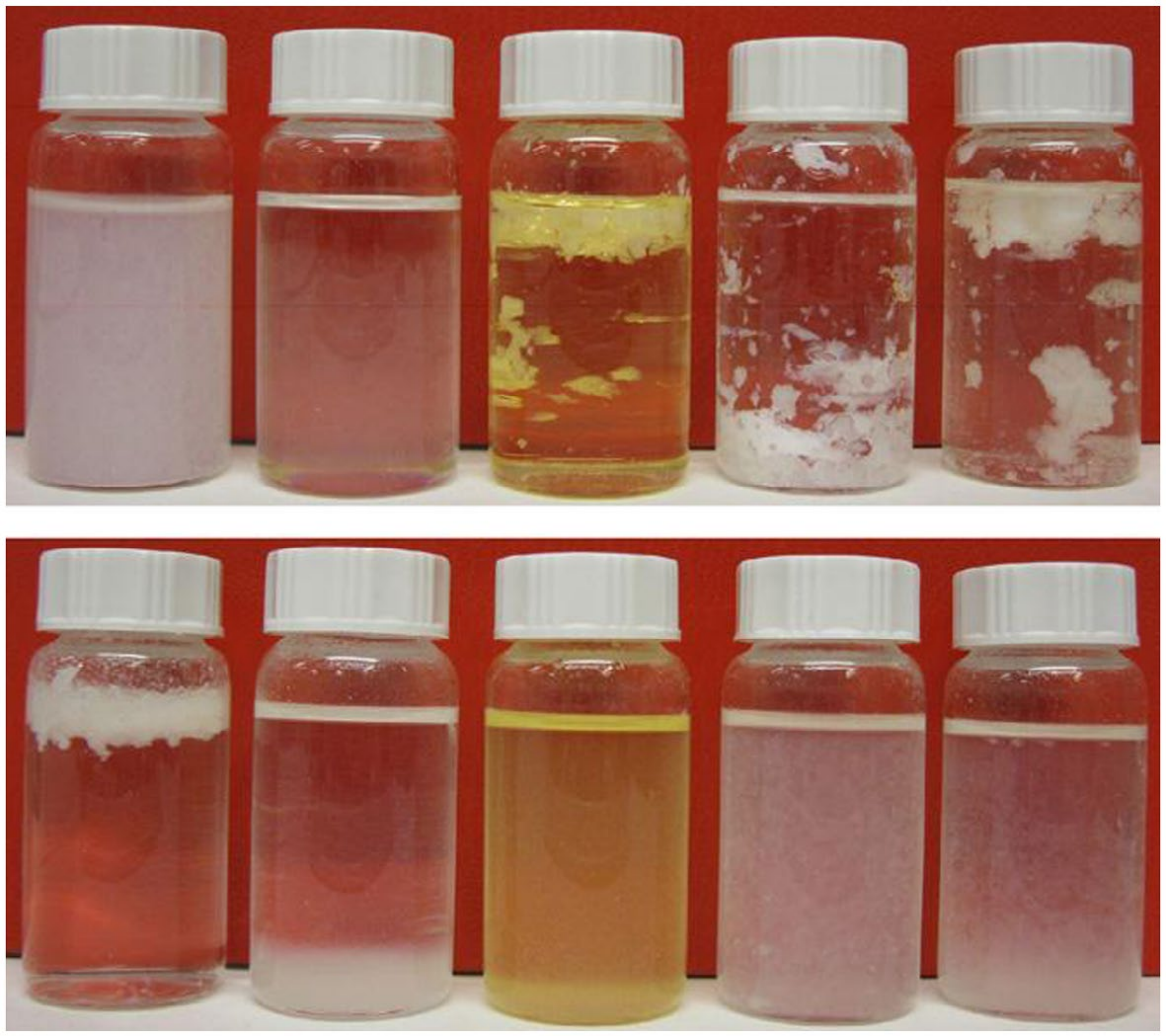
Dispersions of untreated MFC (top image) and MFC-ODA (bottom image) in water, toluene, EU-diesel, dodecane, and FT-diesel (from left to right). Modified from Ref. [89], with permission from Elsevier (© Elsevier 2010).

Hydrophobic/hydrophilic surface properties of nanocellulose may be altered by surface modifications, and suitable surface hydrophobization leads to phase inversion of the Pickering emulsions from o/w to w/o ones. Stenius et al. investigated the stability of w/o emulsions using MFCs hydrophobized with chlorodimethyl isopropylsilane and varying degrees of surface substitution [87, 88]. The hydrophobized MFCs formed stable water-in-toluene emulsions and showed better stability against sedimentation with increasing MFC concentration most likely due to an increase in the viscosity of the continuous oil phase. The hydrophobized MFCs demonstrated optimum substitution conditions for stabilization; a high degree of surface substitution leads to less stability of the emulsions, and they concluded that hydrophobized MFCs with contact angles at ~90° at the interface exhibit good stability, as indicated by a theoretical prediction (Equation 1). A w/o Pickering emulsion of Fischer–Tropsch diesel droplet can be prepared, and optimized surface hydrophobicity of MFCs effectively stabilizes the emulsion (Figure 5) [89].

Lee et al. controlled the surface hydrophobicity of BCNs by changing the grafted chain length (acetic acid, C_2_-; hexanoic acid, C_6_-; dodecanoic acid,C_12_-); the BCNs grafted with long hydrophobic chains, C_6_- and C_12_- acid, form stable w/o Pickering emulsion [90]. Hu et al. finely tailored the emulsion phase by modifying the surfaces with cationic alkyl ammonium surfactants didecyldimethylammonium bromide (DMAB) and cetyltrimethylammonium bromide (CTAB) [91]. The addition of the surfactants was found to increase the emulsion stability and decrease the droplet size. Interestingly, the emulsion showed double phase inversion from o/w to w/o and back to o/w ones with increasing amounts of DMAB; the first phase inversion was caused by the surface hydrophobization of the CNCs by DMAB, and at the second, the stabilization by DMAB became dominant in the system due to the high concentration.

### 2.3. Double emulsions

**Figure 6.**
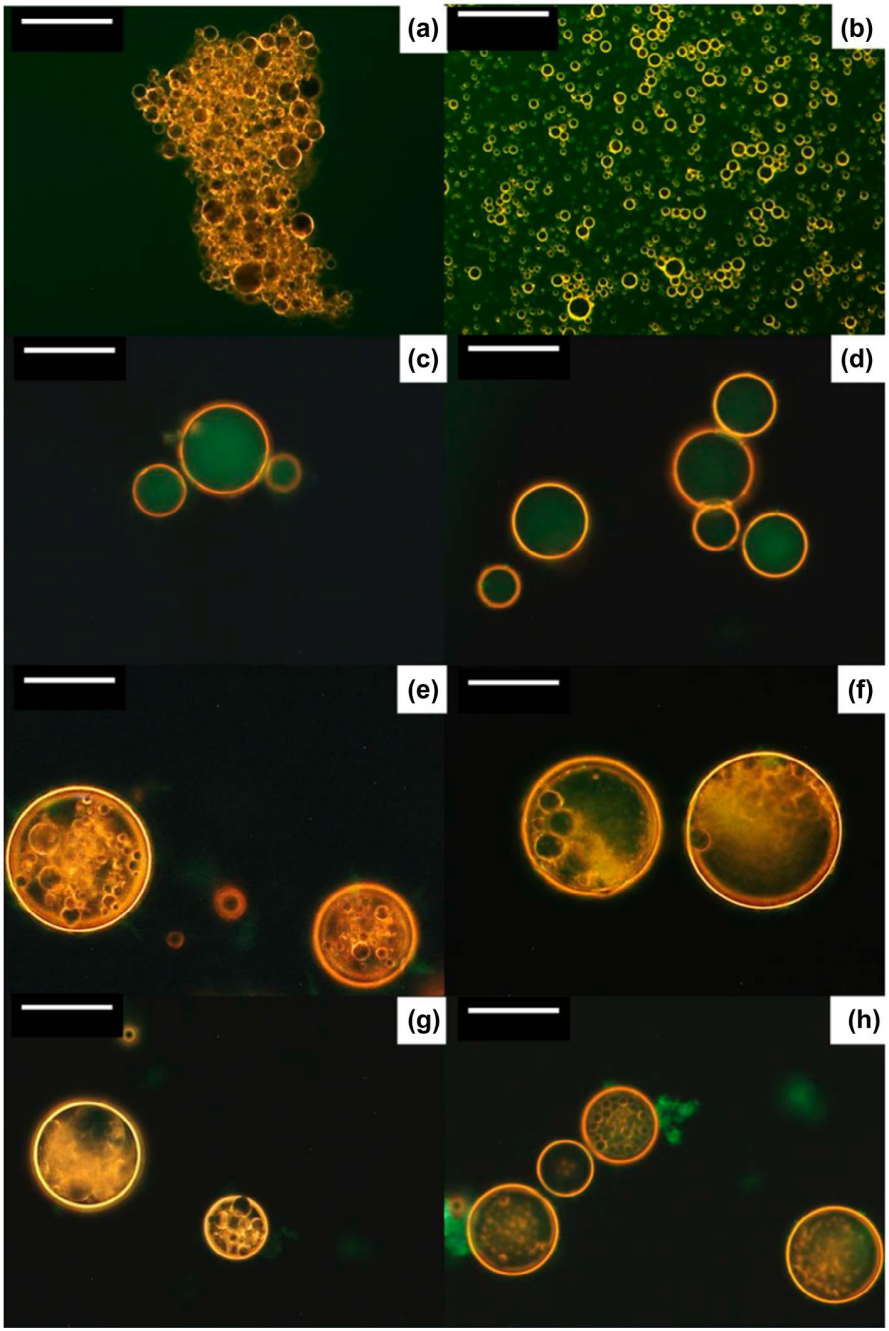
Dark-field microscopy images of o/w emulsions stabilized by (a) NFC and (b) CNC; w/o emulsions stabilized by (c) NFC and (d) CNC modified with lauroyl chloride (C12) (NFCC12 and CNCC12, respectively); and o/w/o double emulsions stabilized by (e) NFC/NFCC12, (f) CNC/NFCC12, (g) NFC/CNCC12, and (h) CNC/CNCC12. Water was stained with fluorescein. Scale bar is 50 μm. Modified from Ref. [78], with permission from American Chemical Society (© ACS 2014).

Double emulsions are complex systems known as an emulsion within an emulsion. Droplets in a dispersed phase contain smaller droplets of different emulsion systems inside. The two major types are oil-in-water-in-oil (o/w/o) or water-in-oil-in-water (w/o/w) emulsions. The systems typically require two different types of surfactants (solid particles for Pickering emulsions): one preferring water and one preferring oil. As described above, the surface of nanocelluloses can be finely tailored by chemical modification, and double emulsion systems can be realized via combinations of unmodified and modified nanocelluloses. An o/w/o double emulsion prepared by a combination of unmodified and chemically modified nanocelluloses was reported by Cunha et al. (Figure 6) [78]. The size of the double emulsion ranged from 43 to 76 μm, in which the o/w emulsion system containing oil droplets of ~3 μm was successfully encapsulated. The o/w/o double emulsion exhibited good stability over a month. Upon centrifugation, the emulsion resisted up to 5000 relative centrifugal forces without disruption. Frank et al. showed that MCCs function as stabilizers in w/o/w emulsions containing oil-soluble surfactants [92, 93].

## 3. Applications

The structural stability and biocompatibility of the nanocellulose-stabilized Pickering emulsions demonstrate potential in drug delivery applications. Jain et al. investigated the drug release rate of MCC-stabilized w/o/w double emulsions, in which MCC acted as a stabilizer for both the internal and external layers [94]. The emulsion showed a slow release rate when the MCC concentration in the internal phase was increased, and this phenomenon was likely due to diffusion control by a viscous MCC network. The release rate was not significantly affected by the pH of the external medium because MCCs show good stability against pH. Moreover, the emulsion showed small drug leakage less than 10% and good stability without any recognizable phase separation upon storage over a period of 90 days. MCC-stabilized w/o/w emulsion also show drug release properties, as reported by Oza and Frank [93]. A novel drug delivery system was developed by Abdalla et al., who mixed a self-emulsifying lipid with MCCs to obtain pellets for improving drug delivery [95]. In the presence of the MCC, the pellets displayed a uniform size and shape and showed good stability.

The o/w Pickering emulsion stabilized by food-grade MCC offers the potential for food application. Kargar et al. investigated the oxidative stability of sunflower oil in the emulsion, and they found out that MCC enhances stability by significantly reducing the lipid oxidation rate; thus, MCC is more effective than modified starch [72]. The charge, size, and concentration of MCC play an important role in the stability. They concluded that this effect can be ascribed to the unique MCC characteristics, such as ability to scavenge free radicals and form a thick layer around oil droplets.

**Figure 7.**
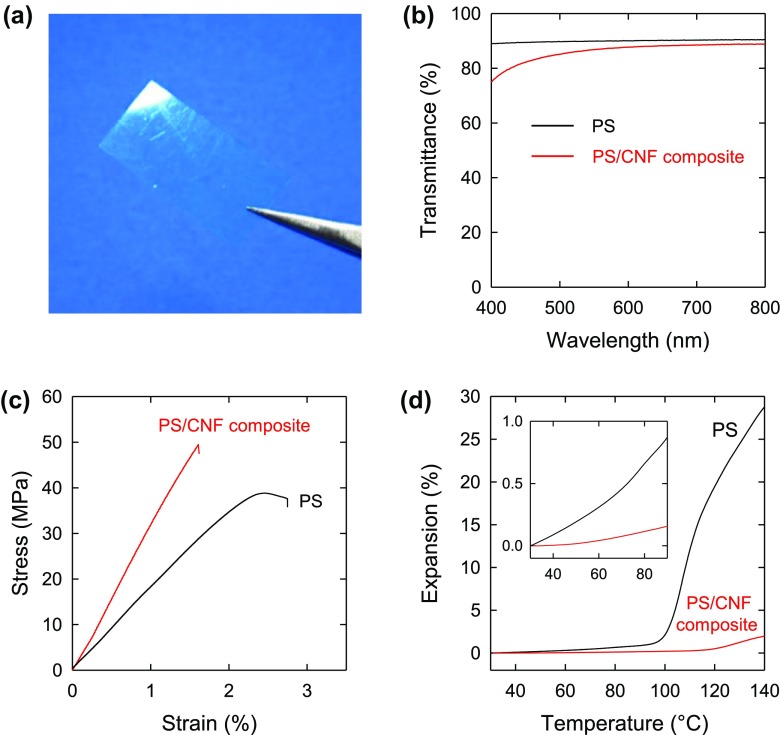
(a) Photograph of polystyrene/nanocellulose composite film with 12% w/w nanocellulose after melt pressing, (b) UV–vis transmittance spectra, (c) stress–strain curves, and (d) thermal expansion behavior of the composite and polystyrene films. Modified from Ref. [74], with permission from American Chemical Society (© ACS 2017).

Nanocellulose/polymer nanocomposite films can be prepared by simply polymerizing monomer droplets of nanocellulose-stabilized o/w Pickering emulsions, which is conducted in a similar way to conventional suspension polymerization. This process is a simple and environmentally friendly aqueous one, in which nanocelluloses work both as stabilizer and nanofillers in the emulsion and polymer composite, respectively. Poly(styrene-*co*-hexylacrylate)/cellulose whisker nanocomposites can be prepared through this process, but a low amount of reactive silane (methacryloxypropyl triethoxysilane) is added to efficiently avoid particle agglomeration [96]; the composite films are obtained by casting and drying the homogeneous dispersion of the cellulose nanowhiskers and polymer particles, and the films show enhanced storage modulus above the glass transition region of the polymer matrix by 500% due to reinforcement by the cellulose nanowhiskers. Gindl-Altmutter et al. used lignocellulose, which is partially delignified wood, as stabilizers [97, 98]. The lignocellulose provided good stabilization of the emulsion system, and this feature was likely due to the enhanced amphiphilic surface chemical character caused by residual hemicellulose and lignin. Transparent nanocellulose/polystyrene nanocomposite films were prepared by hot-pressing treatment after polymerization (Figure 7) [74] using a surface-carboxylated nanocellulose prepared by TEMPO-mediated oxidation as a stabilizer. The mechanical properties were comparable with those of a nanocellulose/polystyrene nanocomposite prepared by solvent casting and drying of nanocellulose/polystyrene mixture in *N,N*-dimethylformamide [99]. Using this process, finely organized nanocellulose/poly(methyl methacrylate) [100] and nano-fibrillated chitin/acrylic resin [101] composites were prepared.

**Figure 8.**
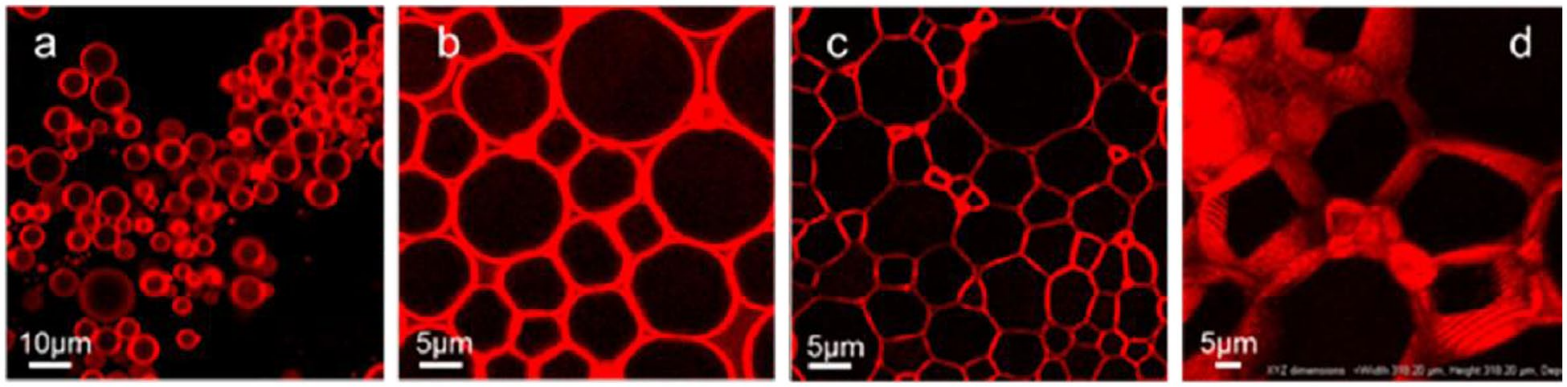
Confocal laser scanning microscopy images of emulsions stabilized by cotton CNCs containing increasing amounts of hexadecane stained with 4,4-difluoro-4-bora-3a,4a-diaza-s-indacene from (a) the original 10/90 oil/water Pickering emulsion; (b) 65% of internal phase; (c) 85.6% of internal phase; (d) the same as panel c using a stacking of 2D 1 μm-thick optical cross-section images to form a 3D reconstruction. Modified from Ref. [76], with permission from American Chemical Society (© ACS 2013).

**Figure 9.**
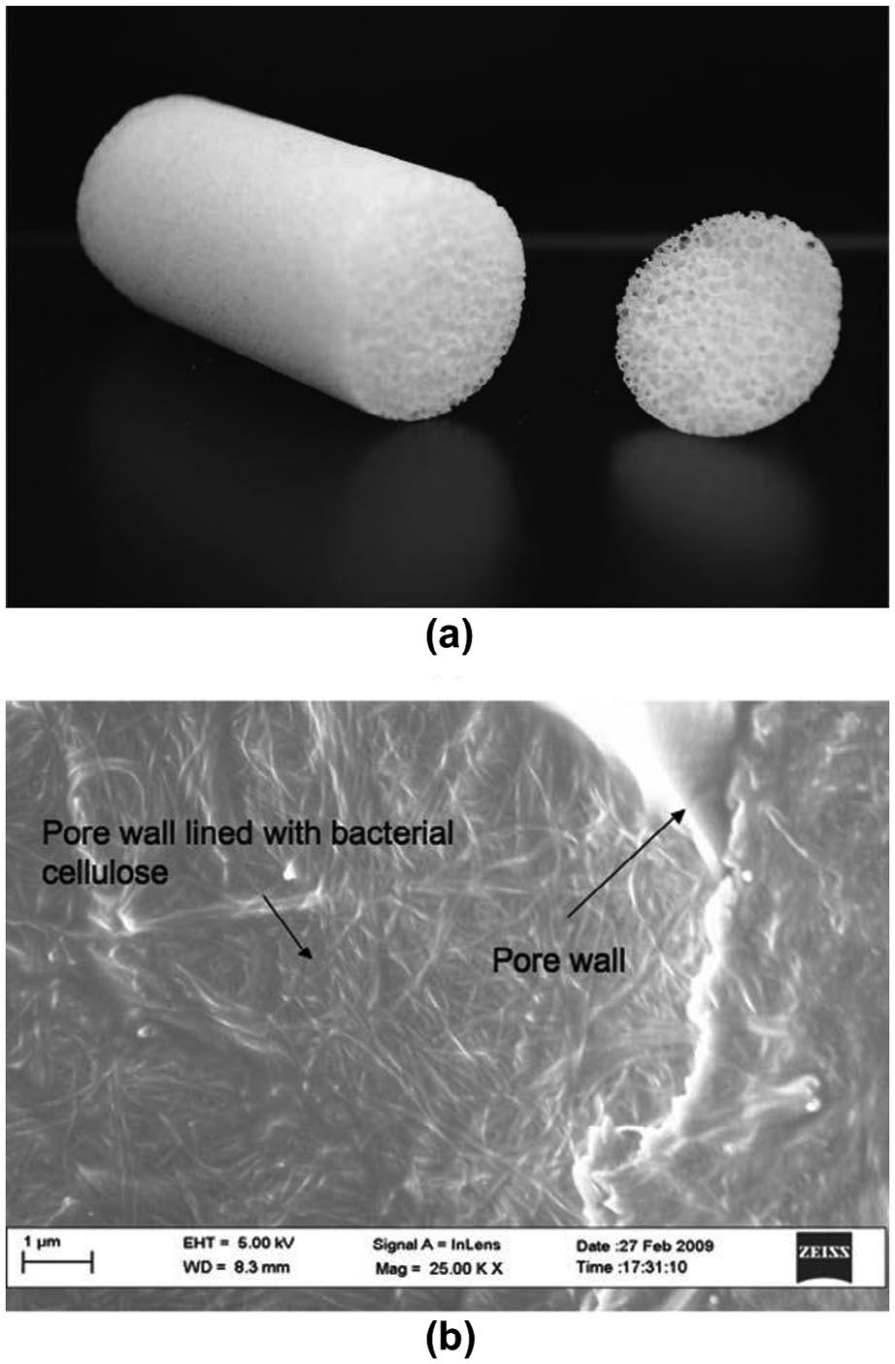
(a) Esterified bacterial cellulose/photopolymerized acrylated epoxidized soybean oil nanocomposite foam (23 mm in diameter). (b) Esterified cellulose nanofibrils are shown to line a pore. Modified from Ref. [105], with permission from Royal Society of Chemistry (© RSC 2009).

Porous and lightweight cellulosic foams can be synthesized by templating the Pickering high internal phase emulsions (HIPEs) [102, 103], in which the stiff network structure of nanocelluloses allows the formation of stable o/w [76] or w/o [90] HIPE systems, with less than 0.1 wt.% of nanocelluloses (Figure 8) [76]. Lightweight cellulosic foams were prepared by simply freeze-drying a CNC-stabilized o/w emulsion, and the entire foam shape and internal structure were preserved due to the excellent mechanical properties of the CNC [104]. The process is facile and easy to handle, and the rigidity of the materials can be controlled by adjusting the concentration of CNCs in the aqueous phase of the starting emulsions. Moreover, further treatment by chitosan mechanically reinforces the foams. Blaker et al. formed a w/o HIPE using a modified soybean oil as the oil phase. After polymerizing the oil phase, porous and fully renewable nanocellulose/polymer composite foams with porosities as high as 76% or 69% were successfully prepared (Figure 9) [105]. Based on the emulsion templating technique, air/water interfaces were also stabilized using surface-hydrophobized nanocelluloses, and lightweight and strong cellulose materials were prepared after drying [106]. In the study, nanocelluloses effectively stabilize the interface and show better stability against coalescence due to their high adsorption energy compared with surfactants. After careful drying under ambient condition, porous cellulose materials with a porosity of 98% and a density of 30 mg cm^−3^ are successfully synthesized, and the materials have high Young’s modulus and compressive energy absorption.

When a magnetically modified CNC (CNCs‒CoFe_2_O_4_) is used as a stabilizer, the CNCs‒CoFe_2_O_4_ densely cover the surface of polymer microbeads, and the hybrid microbeads show favorable magnetoresponsive properties [75]. The microparticles effectively adsorb a cationic dye in water, and they can be removed from media via magnetic separation. Furthermore, CNCs‒CoFe_2_O_4_ hollow microcapsules are produced by dissolving the polymer core. Svagan et al. developed a rapid and facile way to prepare liquid-core capsules with high mechanical stability by covalently cross-linking (by aromatic diisocyanate) the nanocellulose layer of o/w Pickering emulsions [71]. The elastic modulus of the nanocellulose layers increased, and the microcapsules showed enhanced structural stability.

**Figure 10.**
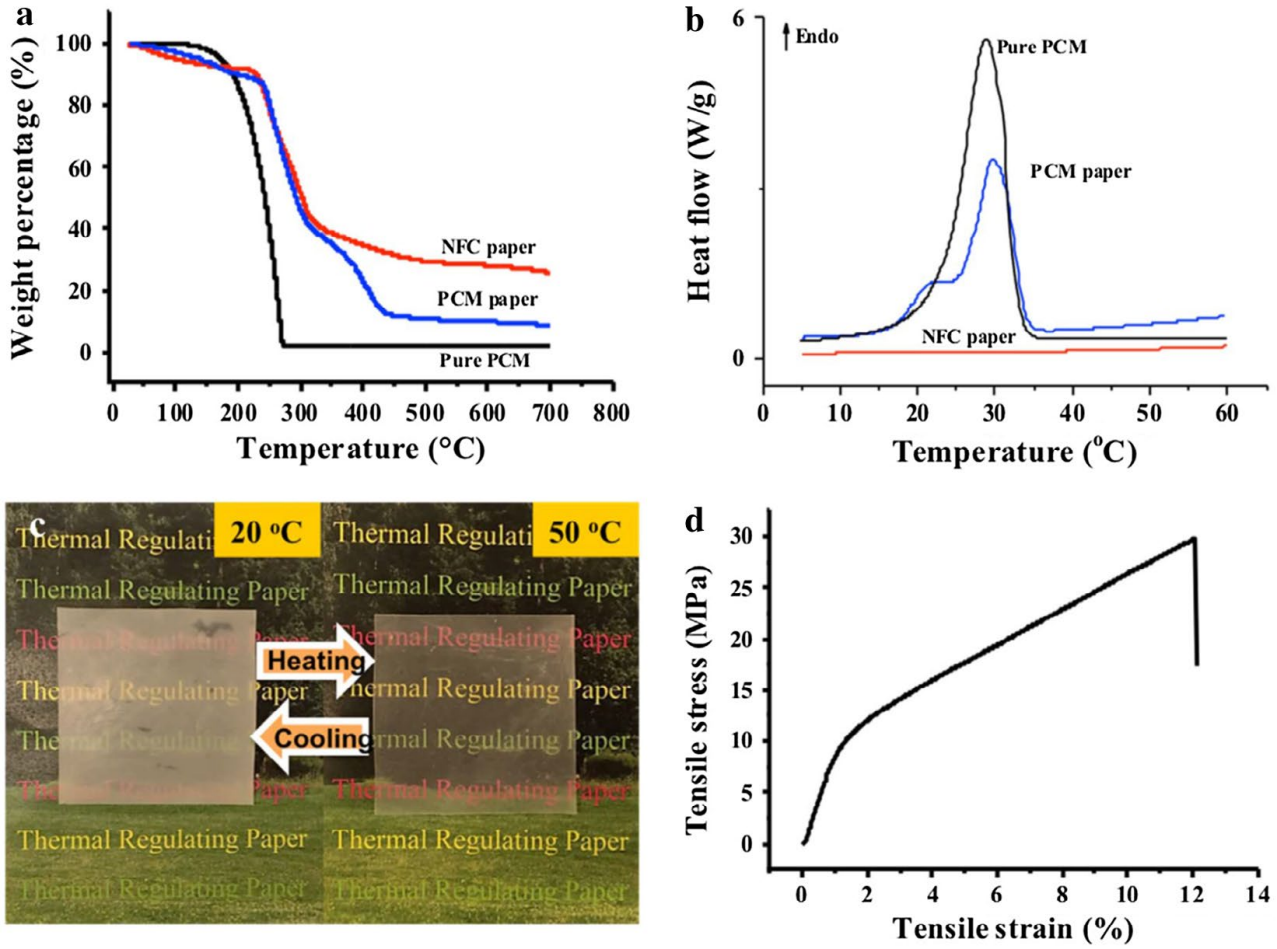
(a) Thermogravimetric analysis and (b) differential scanning calorimetry curves of nanocellulose paper, phase change material (PCM) paper, and pure PCM. c) Optical image of PCM paper during the heat/cool cycles between 20 °C (left) and 50 °C (right) after 10 cycles. d) Stress–strain curve of the PCM paper. Modified from Ref. [83], with permission from Elsevier (© Elsevier 2017).

Li et al. showed a new way to prepare thermal storage materials based on nanocellulose-stabilized o/w Pickering emulsions (Figure 10) [82]. Nanocellulose allowed high content of paraffin encapsulation (more than 72 wt.%) caused by the good emulsifying properties. Upon heating, the encapsulated paraffin underwent phase change with high thermal absorption mainly due to melting of the paraffin, without leakage during heating/cooling cycles. Therefore, the material showed excellent thermal regulation performance.

**Figure 11.**
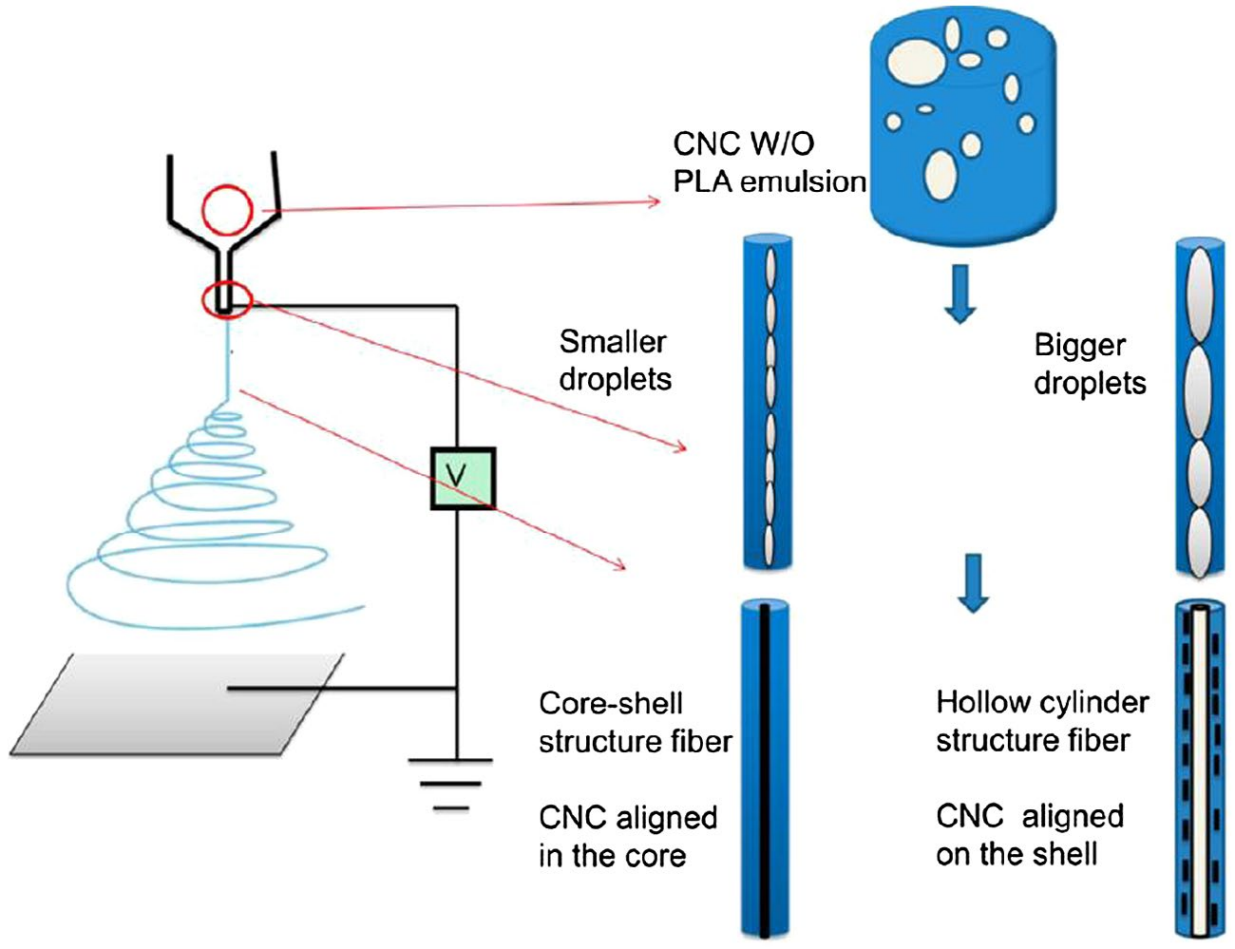
Schematic of emulsion preparation and electrospinning of CNC−poly(lactic acid) (PLA) emulsions. Modified from Ref. [107], with permission from American Chemical Society (© ACS 2013).

Ultrafine CNC-poly(lactic acid) (PLA) composite fibers can be fabricated by electrospinning of a CNC-stabilized w/o emulsion (Figure 11) [107]. Interestingly, the fiber structure can be tuned from a core‒shell/hollow structure to a core‒shell structure by changing the starting emulsion size from larger (average size: 6 μm) to smaller (3 μm) ones, respectively. In the composite fiber, CNCs function as nucleating and reinforcing agents. Therefore, random fiber mats prepared from CNC-PLA exhibit higher Young’s modulus and maximum tensile strength than PLA random fiber mats.

## 4. Future prospects

The excellent stability and biocompatibility of the nanocellulose-stabilized Pickering emulsions offer major advantages in drug delivery or food applications. Given these characteristics, the emulsions can be extended to many other applications such as cosmetics and paints. By templating the unique emulsion structure, well-organized nanocellulose-based polymer composite materials (e.g. films, microparticles, and foams) can be fabricated. These materials combine good mechanical properties and environmentally friendly characteristics. Therefore, they are of great interest in many fields such as food packaging films, separation/purification, and biomedical applications. The main challenge in developing these materials is how to maintain the stability of the Pickering emulsion; there is no sufficient understanding of how a multicomponent solvent system can affect the stability. On the other hand, many fundamental studies on the stability of nanocellulose have been reported, and we expect that the understanding in the field could allow the development of novel Pickering emulsion-based material.

The preparation processes are facile and easy to handle because they are conducted in aqueous solutions, where nanocelluloses are well dispersed and do not require any time-consuming solvent exchanging process. Therefore, material fabrication based on the nanocellulose-stabilized Pickering emulsions paves way toward novel bio-based materials with a facile and scalable process.

## Disclosure statement

No potential conflict of interest was reported by the authors.
